# Delta Neutrophil Index for the Prediction of Prognosis in Acute Gastrointestinal Diseases; Diagnostic Test Accuracy Meta-Analysis

**DOI:** 10.3390/jcm9041133

**Published:** 2020-04-15

**Authors:** Hae Min Jeong, Chang Seok Bang, Jae Jun Lee, Gwang Ho Baik

**Affiliations:** 1 Department of Internal Medicine, Hallym University College of Medicine, Chuncheon 24253, Korea; cromnjeong@hallym.or.kr (H.M.J.); baikgh@hallym.or.kr (G.H.B.); 2 Institute for Liver and Digestive Diseases, Hallym University, Chuncheon 24253, Korea; 3 Institute of New Frontier Research, Hallym University College of Medicine, Chuncheon 24253, Korea; iloveu59@hallym.or.kr; 4 Department of Anesthesiology and Pain Medicine, Hallym University College of Medicine, Chuncheon 24253, Korea

**Keywords:** delta neutrophil index, gastrointestinal diseases, biomarkers, prognosis

## Abstract

Delta neutrophil index (DNI) is a novel diagnostic and prognostic biomarker of various infectious or inflammatory conditions. However, data on optimal measurement time are scarce, and no studies have evaluated the potential role of the DNI as a prognostic biomarker of gastrointestinal diseases with diagnostic test accuracy meta-analysis. Core databases were searched. The inclusion criteria were as follows: patients who have gastrointestinal diseases and DNI measurements presenting diagnostic indices for predicting the prognosis, including severity, surgical outcomes, and mortality from gastrointestinal diseases. We identified twelve studies for the systematic review and ten studies for the quantitative analysis. Pooled area under the curve, sensitivity, specificity, and diagnostic odds ratio of DNI at the initial admission date were 0.82 (95% confidence interval: 0.78–0.85), 0.75 (0.52–0.89), 0.76 (0.63–0.86), and 10 (3–35), respectively. Meta-regression showed no reasons for heterogeneity and publication bias was not detected. Fagan’s nomogram indicated that the posterior probability of ‘poor prognosis’ was 76% if the test was positive, and ‘no poor prognosis’ was 25% if the test was negative. The DNI can be considered as a reliable initial measurement biomarker for predicting prognosis in patients with gastrointestinal diseases,

## 1. Introduction

Gastrointestinal disorders (GI disorders) are associated with substantial morbidity and mortality. These impose a significant healthcare cost worldwide. In Korea, about 13% of the national annual medical spending is attributable to GI disorders [1]. The situation in the US is similar as the annual medical expenditure of GI disorders totaled $135.9 billion in 2015 [2,3]. This amount is greater than that of many common disorders, including traumatic diseases, cardiovascular diseases, and psychiatric illnesses [2,3]. Moreover, these expenditures are expected to continuously rise and the overall visits of emergency centers due to GI disorders have been increasing [1,2,3,4,5]. In the context of the severity of diseases, about one third of hospitalized patients with emergency GI disorders finally needed surgical treatment in the US, indicating the importance of point of care testing and precise triage [6].

Despite recent advances in our understanding of the pathophysiology of various GI disorders, the accurate prediction of disease severity, surgical outcomes, or mortality rates from GI diseases are difficult with an initial measurement of conventional laboratory or radiologic indices. Prognostic predication models have been developed from a combination of ecologic factors, laboratory findings, and clinical factors. However, these models require complex calculations or memorization, and most of them have not been widely validated [3].

DNI is a laboratory index which is calculated as the ratio of immature granulocyte numbers to the total neutrophil counts in peripheral circulation [7]. More specifically, it is calculated by subtracting the fraction of mature polymorphonuclear leukocytes from the sum of myeloperoxidase-reactive cells [7,8]. Because the formation of immature granulocytes precedes changes in total peripheral white blood cell counts in the process of granular leukocyte differentiation of infectious or inflammatory diseases, DNI has received attention as a biomarker for these conditions [8,9].

Previous reports have suggested the beneficial effect of DNI as a diagnostic and prognostic marker of various infectious or inflammatory diseases [8,10]. Sepsis originating from various organs is the most widely studied area for the application of DNI as a prognostic biomarker [8]. However, there are scarce data about optimal measurement time, and no studies have evaluated the potential efficacy of DNI as a prognostic biomarker of GI diseases with diagnostic test accuracy meta-analysis.

Therefore, our study aimed to provide an evidence of DNI as a prognostic biomarker in GI diseases.

## 2. Methods

This diagnostic test accuracy meta-analysis with systematic review was conducted according to the principles of the Preferred Reporting Items for Systematic reviews and Meta-Analyses (PRISMA) [11]. The study protocol was registered at PROSPERO on June 2019 (CRD42019136459) and a study protocol article was also published [3]. 

### 2.1. Literature Searching Strategy

Core database including MEDLINE, EMBASE, and the Cochrane Central Register of Controlled Trials in the Cochrane Library were searched for “delta neutrophil index” (inception to January 2020) by two independent authors (C.S.B. and J.J.L). Only the key phrase, “delta neutrophil index” was searched to maximize the sensitivity of the searching process. We reviewed the abstracts of all the identified articles as the first step of screening. We also conducted full-text reviews as the second step. The references of all the included studies were reviewed to find additional unidentified studies. Disagreements between the evaluators were resolved by discussion or consultation with an independent evaluator (G.H.B.) [3].

### 2.2. Selection Criteria

We included studies that satisfied the following selection criteria: 1. patients: who have GI diseases; 2. intervention: studies with DNI measurements; 3. comparison: none; 4. outcome: diagnostic indices of DNI, such as sensitivity, specificity, positive predictive value (PPV), negative predictive value (NPV), positive likelihood ratio (PLR), negative likelihood ratio (NLR), accuracy, or diagnostic odds ratio (DOR), which enable an estimation of true positive (TP), false positive (FP), false negative (FN), and true negative (TN) numbers for the prediction of prognosis in GI diseases; 5. study design: not limited; 6. studies of adult subjects; and 7. full-text publications in English. Studies that satisfied all of the inclusion criteria were included. The exclusion criteria were as follows: 1. review in narrative format; 2. guideline or consensus document 3. letter, comment, editorial or protocol study; 4. systematic review or meta-analysis. Studies with at least one of the exclusion criteria were excluded [3]. 

### 2.3. Methodological Quality Assessment

The Quality Assessment of Diagnostic Accuracy Studies-2 (QUADAS-2) tool was used for the measurement of methodological quality of all the included studies. This contains four domains: ‘patient selection’, ‘index test’, ‘reference standard’, and ‘flow/timing’ [12]. Each domain was evaluated for the risk of bias, and the first three domains were also evaluated for applicability [12]. Two of the independent authors (C.S.B. and J.J.L.) evaluated the methodological quality of all the included studies, and any disagreements between the evaluators were resolved by discussion or consultation with an independent evaluator (G.H.B.) [3]. Review Manager version 5.3.3 (RevMan for Windows 7, the Nordic Cochrane Centre, Copenhagen, Denmark) was used to create the summary figure of QUADAS-2 results.

### 2.4. Data Extraction, Primary Outcome, and Additional Analyses

Two independent authors (C.S.B. and J.J.L.) used the same fill-in form to collect the primary outcomes (TP, FP, FN, and TN) and modifiers identified in each study. For the studies with incomplete data, we contacted the corresponding author of each study by e-mail to obtain the exact value of TP, FP, TN, and FN. Disagreements between the two evaluators were resolved by discussion or consultation with an independent evaluator (G.H.B.). 

Diagnostic test accuracy was the primary outcome of this study. We calculated the crude values of TP (subjects with a (+) DNI who have a poor prognostic form of GI diseases), FP (subjects with a (+) DNI who do not have a poor prognostic form of GI diseases), FN (subjects with a (−) DNI who have a poor prognostic form of GI diseases), and TN (subjects with a (−) DNI who do not have a poor prognostic form of GI diseases) of DNI for the prediction of prognosis in GI diseases in each study. We used 2 × 2 tables containing various diagnostic performance indices from the original studies whenever possible. If only a part of the data was presented, authors calculated the crude values of TP, FP, FN, and TN as previously described [3,13].

The following modifiers were also extracted, whenever possible: format of study, age, sex, ethnicity, enrolled number of patients, publishing year, method of measurement in DNI, cut-off value of DNI, and type of prognosis or form of GI diseases [3].

### 2.5. Statistics

The Stata Statistical Software, version 15.1 (Stata Corp, College Station, TX, USA) with Metandi and Midas packages was used for the statistical analyses. A narrative summary was conducted through the systematic review and meta-analysis (bivariate random model) [14], and the hierarchical summary receiver operating characteristic (HSROC) model [15]) was also used. A Forest plot of sensitivity and specificity (a bivariate model) and summary receiver operating characteristic (SROC) curve (a HSROC model) were presented. Heterogeneity across the studies was evaluated by correlation coefficient between specificity and logit transformed sensitivity (a bivariate model) [14] and asymmetry parameter, β (beta) (β = 0 corresponds to a symmetric receiver operating characteristic (ROC) curve, in which the DOR does not vary along the curve by HSROC model) [15,16]. A correlation coefficient greater than zero, and β with P value less than 0.05 indicates heterogeneity across studies [15,16]. Visual inspection of the SROC curve was also conducted to find heterogeneity. Subgroup analysis was also performed to check robustness of the results, and meta-regression with the appropriate modifiers was conducted to find the reason for heterogeneity. Publication bias was assessed through the asymmetry test of Deeks’ funnel plot [3].

## 3. Results

### 3.1. Study Selection

**Figure 1:**
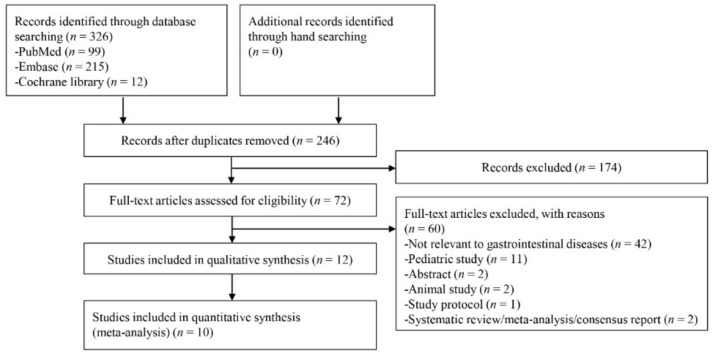
Flow diagram of study selection.

In total, 326 studies were identified by searching the three databases. 80 duplicate articles and 174 additional articles were excluded through screening by reviewing titles and abstracts as the first step. The remaining 72 studies were reviewed through full-text reading. 60 studies were finally excluded according to the exclusion criteria: not relevant to GI diseases (*n* = 42), pediatric study (*n* = 11), abstract only study (*n* = 2), animal study (*n* = 2), study protocol (*n* = 1), and systematic review or meta-analysis (*n* = 2). The remaining 12 articles [9,17,18,19,20,21,22,23,24,25,26,27] were included in the systematic review. Among the 12 studies, Kim JW et al. [22] explored the prognostic role of DNI measured at admission day 3, and the results of the study by Cha YS et al. [26] cannot be extrapolated as a form of TP, FP, FN, or TN (although we contacted corresponding author, we could not get the crude numbers of TP, FP, FN, or TN). Therefore, these two publications were excluded in the quantitative analysis, and a total of 10 studies [9,17,18,19,20,21,23,24,25,27] were included in the meta-analysis of diagnostic test accuracy. Figure 1 presents a flow diagram showing the process for identifying the relevant studies.

### 3.2. Study Characteristics

From the 10 studies [9,17,18,19,20,21,23,24,25,27] for the prediction of prognosis in GI diseases, a total of 2766 patients (518 patients with GI diseases vs. 2248 controls) were identified. Among all, eight studies [9,17,18,19,20,21,23,27] were conducted in a case-control format, whereas two studies [24,25] were conducted in a cohort format. All the studies were conducted in Korea, and the median age of the population enrolled in the studies ranged from 35 to 70 years. Male predominance was commonly detected, except for only one study [19]. To measure the DNI, all the enrolled studies commonly used ADVIA 120/2120/2120i^®^ (Siemens Healthcare Diagnostic Inc., Tarrytown, NY, USA) (Table 1). These characteristics (modifiers) were evaluated as a potential source of heterogeneity through the meta-regression and subgroup analysis.

### 3.3. Quality of the Methodology

Among the 12 studies in the systematic review, 8 publications showed low risk of bias, and 11 studies showed low applicability concerns. 

Three studies [20,21,26] specified need for surgery as a reference standard of positive DNI, which is not an objective indicator. Therefore, these three publications were rated as ‘high-risk’ in the ‘reference standard’ domain. Among the enrolled studies, only the study by Kim JW et al. [22] measured DNI at day 3 and assessed the predictive role of DNI for 30-day mortality after emergent surgery due to acute peritonitis. Because the time interval between index test and reference standard was not suitable, unlike in other studies, this study was rated ‘high-risk’ in the ‘flow/timing’ domain. 

In terms of applicability concerns, the study by Cha YS et al. [26] assessed the prognostic role of DNI for predicting the need for surgery in patients with strangulated mechanical bowel obstruction. However, these patients do not match DNI applicability, because most patients with strangulated mechanical bowel obstruction required surgical treatment.

**Figure 2:**
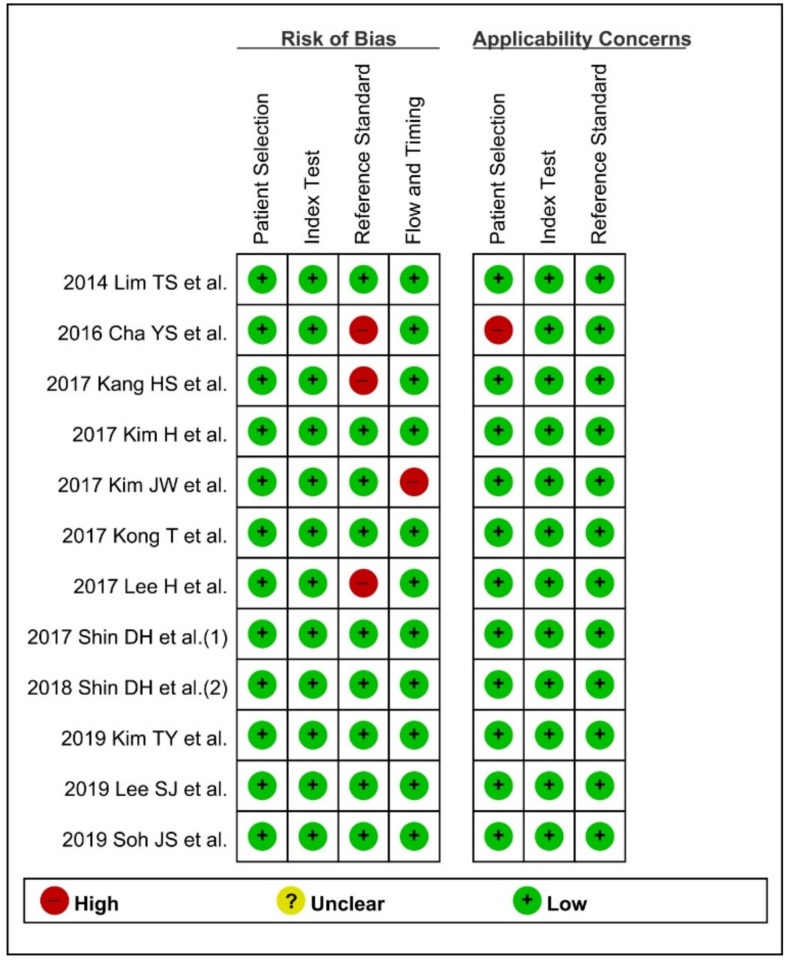
Quality Assessment of Diagnostic Accuracy Studies-2 for evaluation of methodology quality. (+) denotes low risk, (−) denotes high risk.

These four studies with ‘high-risk’ in at least one of the 7 domains were evaluated as low methodological quality for the subgroup analysis (Figure 2).

### 3.4. Diagnostic Indices of DNI for the Prediction of Prognosis of GI Diseases

**Figure 3:**
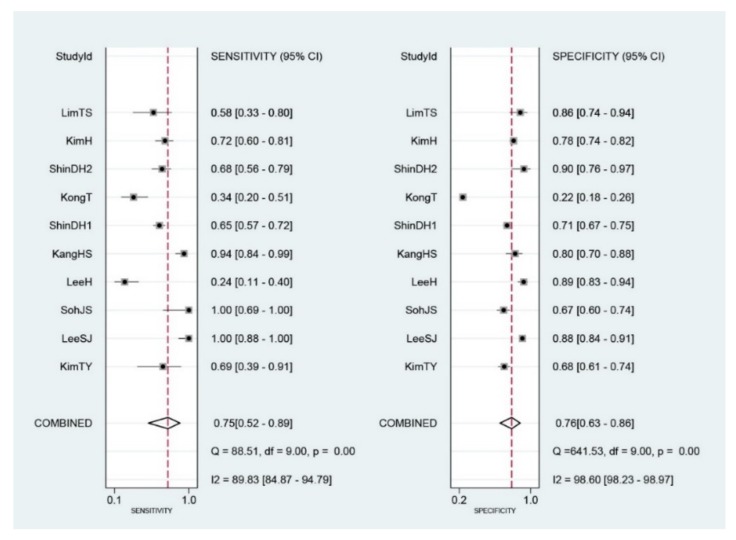
Forest plots of sensitivity and specificity of initial Delta neurophil index (DNI) for the prediction of poor prognosis in gastrointestinal diseases. The diamond indicates the combined estimate from the included studies.

**Figure 4:**
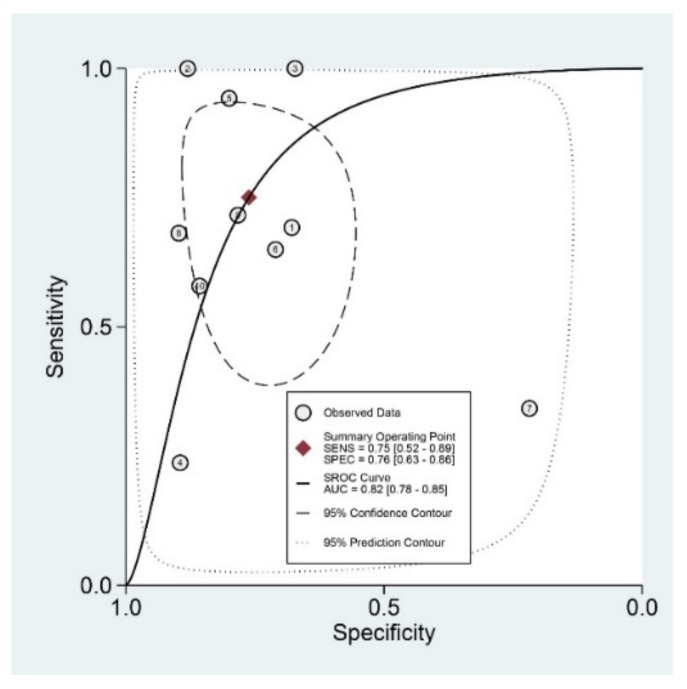
Summary receiver operating characteristic curve with a 95% confidence region and a prediction region for the prediction of poor prognosis in gastrointestinal diseases. SENS, sensitivity; SPEC, specificity; SROC, summary receiver operating characteristic; AUC, area under the curve.

**Figure 5:**
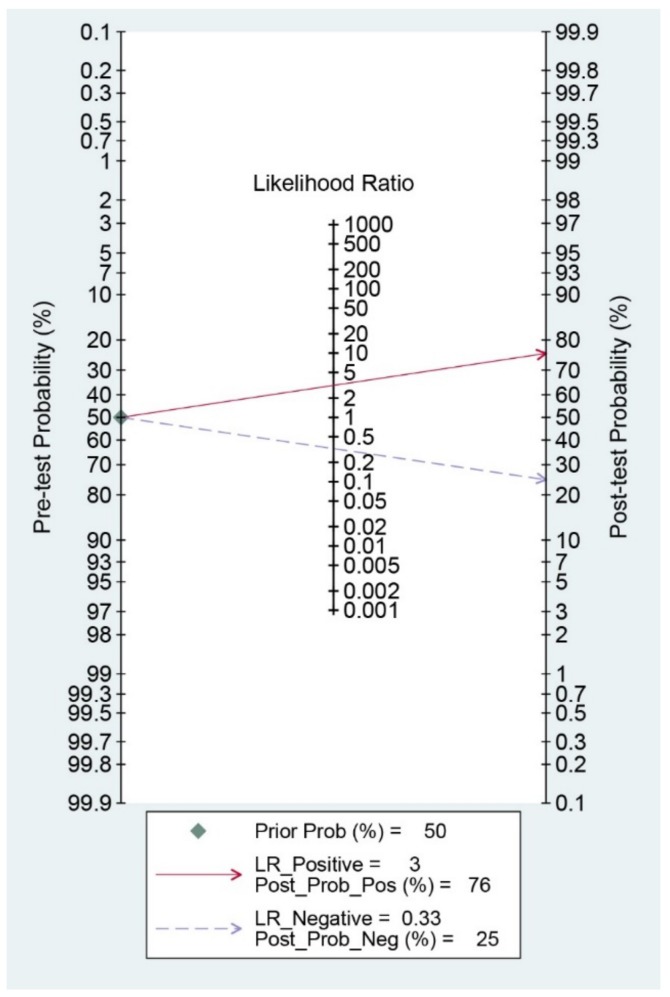
Fagan’s nomogram for the prediction of poor prognosis in gastrointestinal diseases.

Diagnostic indices for the prediction of prognosis of GI diseases, including sensitivity, specificity, PLR, NLR, DOR, and area under the curve (AUC) with 95% CI of DNI for the prediction of prognosis of GI diseases were 0.75 (95% CI: 0.52–0.89), 0.76 (0.63–0.86), 3.1 (1.8–5.6), 0.33 (0.15–0.74), 10 (3–35), and 0.82 (0.78–0.85), respectively (Table 2, Figure 3). Figure 4 shows the SROC curve with a 95% confidence region and a prediction region. Assuming 50% prevalence of the severe form of GI diseases among all the visited patients at emergency department (prior probability), Fagan’s nomogram indicates that the posterior probability of ‘poor prognosis’ was 76% if the test was positive, and the posterior probability of ‘no poor prognosis’ was 25% if the test was negative (Figure 5).

### 3.5. Meta-Regression and Subgroup Analysis 

For the prediction of poor prognosis in patients with GI diseases, the SROC curve was generated, and the shape of the curve was symmetric (Figure 4). We observed an asymmetric index, β, which was not statistically significant (*p* = 0.25), suggesting there was no heterogeneity across the studies. Although the area of 95% prediction region seems a little wide in the SROC curve, meta-regression showed no reasons for the heterogeneity (publishing year (*p* = 0.22), number of enrolled subjects in each study (*p* = 0.39), cut-off value of DNI in each study (*p* = 0.09), age (*p* = 0.84), gender (*p* = 0.51), study format (*p* = 0.09), quality of methodology (*p* = 0.49), surgical or medical condition (*p* = 0.17). Subgroup analyses revealed higher AUCs or DORs in studies with a younger population (<60 years), case-control format, recent publication, low number of included patients (<200), and surgical conditions (Table 2).

### 3.6. Publication Bias

**Figure 6:**
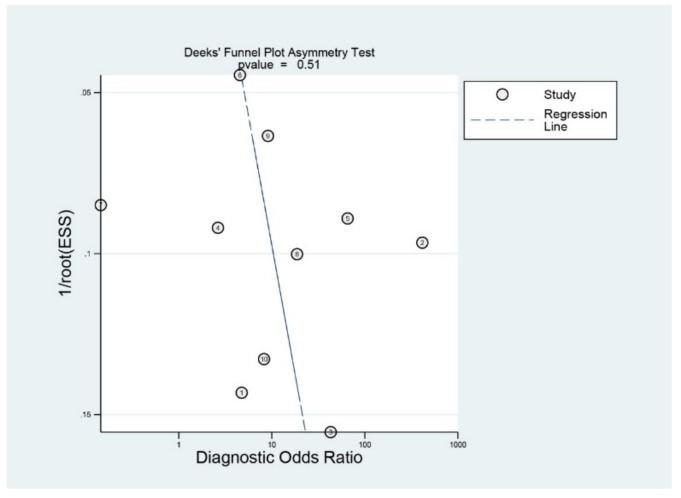
Deek’s funnel plot.

Figure 6 shows the funnel plot. The shape of the plot was grossly symmetrical with respect to the regression line. Deeks’ funnel plot asymmetry test also showed a statistically non-significant *p* value (*p* = 0.51), indicating no evidence of publication bias.

## 4. Discussion

Migration of neutrophils to the target organ is limited by the overproduction of cytokines and chemokines in the early stage of infection or inflammation [28]. Therefore, less mature neutrophils enter into the circulation to compensate for the deficiency of active neutrophils (so-called “left-shift”) [28]. The concept of using the number of immature granulocytes to predict severe infection or inflammation necessitates measuring them manually [29]. However, it is very difficult to accurately and reliably estimate their number. DNI reflecting the ratio of immature granulocyte to the total neutrophil count can be automatically measured with myeloperoxidase and nuclear lobularity channels; this makes predicting the prognosis of various infectious or inflammatory conditions easy and reproducible [10]. Moreover, it indicates immature granulocyte count in the circulation irrespective of the total leucocyte count [8].

Biomarkers are defined as an objectively measured and evaluated indicator reflecting normal biologic or pathologic processes for the determination of diagnosis, prognosis or therapeutic interventions [30,31]. A previous meta-analysis assessed the role of DNI as a prognostic marker for mortality in sepsis [8]. However, the primary concern of this study was the relation between elevated DNI and mortality in patients with sepsis. The diagnostic test accuracy was calculated based on only four studies, which limit the generalization. Various cut-off values of DNI in each of the publications were not considered as a threshold effect. Another previous meta-analysis assessed the performance of DNI as a diagnostic and prognostic marker of infection [10]. The sensitivity and specificity for infection determination were 67% and 94%, respectively, and 70% and 78% for prognosis determination, respectively [10]. However, only a bivariate model for diagnostic test accuracy was considered, and the enrolled publications were not limited to emergent conditions with GI disorders. Furthermore, the measurement time of DNI was not uniform in the included studies [8] and, in some cases, it was not considered in the analysis [10]. 

The results of our study indicate that the performance of DNI is useful for the prediction of severity, surgical outcomes, or mortality rates in patients with GI diseases, considering its high sensitivity, specificity, and AUC (Table 2). Another finding of this study is that the measurement time of DNI at the date of initial admission (or at an emergency department) could be useful as a triage tool for patients with emergent GI diseases. Through the thorough systematic review process, authors only included studies that measured the DNI at the initial admission date in the diagnostic test accuracy meta-analysis. Another important implication would be that the diagnostic validity of DNI is high enough, irrespective of the types of GI diseases. Although previous meta-analyses commonly indicated the usefulness of DNI in patients with infection or sepsis [8,10], these studies did not consider or perform separate analysis confined to GI diseases. The cut-off value of DNI varied in the systematic review process, ranging from 0.7% to 5.7% (Table 1). However, this was not a statistically significant modifier associated with heterogeneity in the meta-regression analysis.

Although, this study rigorously evaluated the performance of DNI, excluding threshold effect, there are several inevitable limitations originating from the potential bias in each study. Firstly, we could only obtain results from one time point measurement of DNI. C-reactive protein (CRP), which is a well-known indicator of various infectious or inflammatory conditions, enhances Toll-like receptor-mediated superoxide release from neutrophils, potentially increasing oxidative stress, but also protecting the host from infection [32]. As the half-life of CRP is about 19 h, which is long enough for the steady time course [33,34], its repeated measurement has been generally used despite considerably varying data regarding its diagnostic accuracy as a marker in predicting bacterial infections [35]. Procalcitonin is another widely-used biomarker that is undetectable in subjects without infection, but it is upregulated by cytokines released in response to bacterial infections [36]. Its half-life is about 25–30 h, and its level declines rapidly with the resolution of inflammation [36]. Unlike these two representative biomarkers of infectious or inflammatory conditions, DNI has a relatively shorter half-life of 3 h and comparable diagnostic validity (recent diagnostic meta-analysis revealed that pooled sensitivity, specificity, and AUC of procalcitonin for the diagnosis of sepsis was 77%, 79%, and 0.85, respectively [37]). Therefore, DNI seems to readily and reliably reflect the status of the patients [10]. However, there have been scarce data about the serial measurement of DNI, and only a recently published study investigated the usefulness of repetitive measurement in patients with acute poisoning [38]. Secondly, there was a slight change in diagnostic indices according to the modifiers in the subgroup analyses of DNI (Table 2). These modifies include a younger population (<60 years), case-control study format, recent publication, and low number of included patients (<200). Included studies with these modifiers commonly demonstrated higher AUCs or DORs in the subgroup analysis. However, these modifiers were not a cause of heterogeneity in meta-regression, and the difference between diagnostic values of subgroup analysis and the absolute value of meta-analysis of all the included studies was not substantial (Table 2). Because all the included studies were conducted in Korea, and no studies confirmed the diagnostic validity of DNI as an external validation, further studies are needed to understand the rationale for widespread use of this biomarker. However, considering the non-invasive nature and easily interpretable characteristics, the parameters revealed in this study suggest the substantial utility of DNI as an initial triage tool for the predication of severe GI diseases.

In conclusion, DNI can be considered as a reliable initial measurement biomarker for predicting the prognosis of GI diseases including their severity, surgical outcomes, or mortality rates.

## Figures and Tables

**Table 1:** Summary of characteristics of included publications.

Study	Diagnosis	Number of Patients	Number of Controls	Cut-off Value	Age (years, Mean ± SD)	Gender (M/F)	Multivariate Analysis (OR, (95% CI))	TP	FP	FN	TN
Kim TY et al. (2019) [9]	Severe acute pancreatitis	13	196	1.8%	Median 65 (IQR: 50–78)	119/90	1.122 (1.045–1.205)	9	63	4	133
Lee SJ et al. (2019) [17]	Severe acute cholecystitis	28	351	3.5%	Median 62 (IQR: 52–73)	214/165	1.97 (1.50–2.60)	28	42	0	309
Soh JS et al. (2019) [18]	30-day mortality after emergent surgery due to acute peritonitis	10 (mortality)	174 (non-mortality)	0.9%	Mortality: 59.1 ± 20.7, non-mortality: 53.5 ± 18.1	118/66		10	57	0	117
Shin DH et al. (2018) [19]	Acute perforated appendicitis (vs. non-perforated appendicitis)	69	39	1.4%	Median 72 (IQR: 67–77)	42/66	9.38 (2.51–35)	47	4	22	35
Lee H et al. (2017) [20]	Need for surgery in patients with intestinal obstruction	38	133	4.3%	Median 60 (range: 20–90)	87/84	3.092 (1.072–8.918) (cut-off 4.3% standard)	9	14	29	119
Kang HS et al. (2017) [21]	Need for surgery in patients with acute colonic diverticulitis	52	80	0.7%	Median 56 (IQR: 41–71)	72/60	1.664 (1.203–2.301)	49	16	3	64
Kim JW et al. (2017) [22]	30-day mortality after emergent surgery due to acute peritonitis	44 (mortality)	116 (non-mortality)	7.8% (at day 3)	Median 70 (IQR: 59–79)	96/64	1.286 (1.145–1.444)	34	5	10	111
Shin DH et al. (2017) [23]	Acute complicated appendicitis (vs. non-complicated appendicitis)	177	438	0.6%	Median 35 (IQR: 23–48)	320/295	4.10 (2.94–5.80)	115	127	62	311
Kong T et al. (2017) [24]	30-day mortality in patients with upper gastrointestinal hemorrhage	38 (mortality)	394 (non-mortality)	1% (at admission)	63.9 ± 14.4	297/135	HR: 1.09 (1.019–1.166)	13	308	25	86
Kim H et al. (2017) [25]	Shock requiring vasopressor or inotrope in acute cholangitis	74	387	4.3%	66.22 ± 13.02	253/208	1.103 (1.045–1.164)	53	84	21	303
	28-day mortality in acute cholangitis	17	444	4.7%			HR: 1.102 (1.053–1.153)	12	111	5	333
Cha YS et al. (2016) [26]	Need for surgery in patients with strangulated mechanical bowel obstruction	15	145		Median 69 (IQR: 54–76)	96/64					
Lim TS et al. (2014) [27]	30-day mortality in patients with spontaneous bacterial peritonitis	19	56	5.7%	Median 59 (range: 38–82)	65/10		11	8	8	48

DNI, delta neutrophil index; SD, standard deviation; M, male; F, female; OR, odds ratio; CI, confidence interval; TP, true positive; FP, false positive; FN, false negative; TN, true negative.

**Table 2:** Summary of outcome of diagnostic accuracy and subgroup analyses.

Subgroup	Number of Included Studies	Sensitivity (95% CI)	Specificity (95% CI)	PLR	NLR	DOR	AUC
Value of meta-analysis in all the included studies	10	0.75 (0.52–0.89)	0.76 (0.63–0.86)	3.1 (1.8–5.6)	0.33 (0.15–0.74)	10 (3–35)	0.82 (0.78–0.85)
Age (years, median or mean)							
<60	4	0.85 (0.54–0.96)	0.75 (0.67–0.81)	3.4 (2.4–4.6)	0.20 (0.06–0.74)	16 (4–74)	0.81 (0.77–0.84)
60≤	6	0.67 (0.36–0.88)	0.76 (0.54–0.90)	2.8 (1.0–7.5)	0.43 (0.16–1.15)	6 (1–42)	0.78 (0.75–0.82)
Quality of methodology of included studies							
High-quality	8	0.73 (0.53–0.86)	0.73 (0.57–0.84)	2.7 (1.4–5.2)	0.38 (0.18–0.78)	7 (2–27)	0.79 (0.75–0.82)
Low-quality	2	Null	Null	Null	Null	Null	Null
Gender							
Male-predominant	9	0.77 (0.50–0.92)	0.74 (0.60–0.85)	3.0 (1.6–5.5)	0.31 (0.12–0.83)	10 (2–43)	0.81 (0.78–0.84)
Female-predominant	1	Null	Null	Null	Null	Null	Null
Format of study							
Case-control	8	0.81 (0.53–0.94)	0.81 (0.73–0.87)	4.2 (2.9–6.2)	0.24 (0.08–0.67)	18 (5–65)	0.86 (0.82–0.88)
Cohort	2	Null	Null	Null	Null	Null	Null
Published year							
Before 2018	6	0.61 (0.37–0.81)	0.74 (0.53–0.87)	2.3 (1.0–5.4)	0.53 (0.26–1.06)	4 (1–19)	0.73 (0.69–0.77)
After 2018	4	0.93 (0.47–0.99)	0.79 (0.66–0.88)	4.5 (2.5–7.9)	0.09 (0.01–1.14)	53 (3–954)	0.88 (0.85–0.91)
Total number of included subjects							
<200	5	0.81 (0.39–0.97)	0.83 (0.74–0.89)	4.7 (3.5–6.2)	0.22 (0.05–0.99)	20 (4–95)	0.90 (0.87–0.92)
200≤	5	0.66 (0.47–0.81)	0.68 (0.44–0.85)	2.0 (0.8–5.1)	0.51 (0.22–1.15)	4 (1–23)	0.72 (0.67–0.75)
Surgical condition vs. medical condition							
Surgical condition	6	0.87 (0.49–0.98)	0.82 (0.73–0.88)	4.8 (3.2–7.4)	0.15 (0.03–0.86)	31 (5–214)	0.87 (0.84–0.90)
Medical condition	4	0.60 (0.43–0.75)	0.64 (0.37–0.85)	1.7 (0.6–4.4)	0.62 (0.29–1.36)	3 (0–15)	0.65 (0.60–0.69)

CI, confidence interval; PLR, positive likelihood ratio; NLR, negative likelihood ratio; DOR, diagnostic odds ratio; AUC, area under the curve.
